# Life-Threatening Cardiac Tamponade Caused by Perforation of an Accessory Left Atrial Appendage During Mitral Isthmus Ablation

**DOI:** 10.1016/j.cjco.2025.01.011

**Published:** 2025-01-17

**Authors:** Chisa Asahina, Kenji Kuroki, Yuya Tanaka, Koji Sudo, Shigeaki Kaga, Akira Sato

**Affiliations:** a Department of Cardiovascular Medicine, Faculty of Medicine, University of Yamanashi, Yamanashi, Japan; b Department of Cardiovascular Surgery, Faculty of Medicine, University of Yamanashi, Yamanashi, Japan

**Figure 1:**
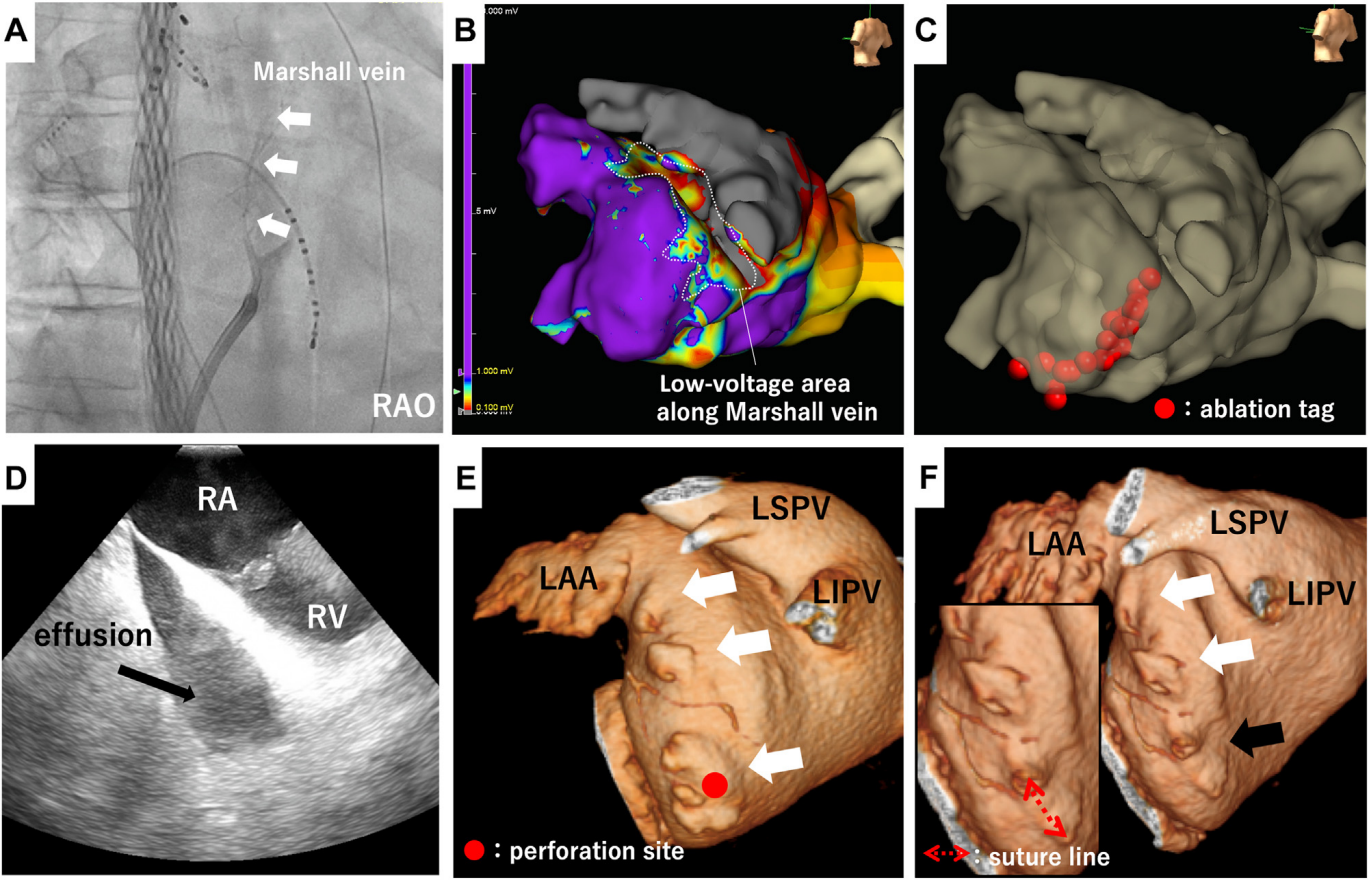
(**A**) Imaging of the Marshall vein before ethanol injection (**white arrows**) in an RAO view. (**B**) Voltage map after ethanol injection into the Marshall vein. A low-voltage area is observed along the ridge between the LAA and left pulmonary veins. (**C**) Ablation line on the mitral isthmus. (**D**) Pericardial effusion observed on intracardiac echocardiography. (**E**) 3D-CT image around the mitral isthmus before ablation, showing multiple accessory LAAs (**white arrows**). (**F**) 3D-CT image around the mitral isthmus after ablation. The largest LAA appears to have been sutured during the cardiac surgery (**black arrow**). 3D-CT, 3-dimensional computed tomography; LAA, left atrial appendage; LIPV, left inferior pulmonary vein; LSPV, left superior pulmonary vein; RA, right atrium; RAO, right anterior oblique; RV, right ventricle.

A 75-year-old male patient underwent a second catheter ablation to treat persistent atrial fibrillation. Three-dimensional electroanatomic mapping (Ensite X; Abbott Laboratories, Chicago, IL) was performed using a multipolar catheter (Advisor HD grid; Abbott) and an SL0 sheath (10.5 Fr; St Jude Medical, St Paul, MN), and radiofrequency application was delivered using a flex-tip ablation catheter (TactiFlex SE; Abbott) and a deflectable sheath (Agilis NxT; Abbott). In addition to bilateral pulmonary vein re-isolation after electroanatomic mapping around the pulmonary veins, a mitral isthmus (MI) block was created. A total of 5 mL of ethanol was injected into the Marshall vein (Fig. 1, A and B), followed by linear ablation from the mitral annulus to the left inferior pulmonary vein using 40 W power and 30-second duration for each point (Fig. 1C). No impedance rise or steam pops were observed; however, at the end of the procedure, the patient’s blood pressure decreased to 66/45 mm Hg and intracardiac-echocardiography revealed cardiac tamponade with massive pericardial effusion (Fig. 1D). Despite draining 2000 mL of blood after pericardiocentesis, the bleeding did not stop and an emergency open-chest surgery was performed. It was difficult to stop the bleeding with beating, but with on-pump, the perforation site was identified on the left atrium near the posterolateral side of the mitral valve and was successfully repaired by suturing with an autologous pericardial strip. Initially, it was assumed that the perforation site was in the low-potential area along the Marshall vein, but it was actually located far away from that area. Looking back at the pre-ablation computed tomography (CT), multiple accessory left atrial appendages (LAAs) with irregular contours were present along the lateral mitral annulus (Fig. 1E, white arrows). Whether injection of ethanol in the vein of Marshall played a role in the complicating perforation of the atrium is unknown, but postoperative CT confirmed that the perforation site was consistent with the repaired accessory LAA (Fig. 1F, black arrow).

The prevalence of accessory LAA is reported to be 5% to 6%,^1^ which is increasingly recognized because of recent improvements in CT resolution. Left atrial diverticulum typically has a sac-like, smooth body with a broad-based ostium, whereas accessory LAA can be identified by its ostium associated with a visible neck and a body with irregular contours that are continuous with the pectinate muscles.^1^ Both are prone to rupture in catheter-based procedures and, rarely, thrombus formation. Detailed preoperative CT review is crucial, allowing consideration of alternative lines, such as the anterior line, if the accessory LAAs are present on the MI. In this case, we did not observe the MI using intracardiac-echocardiography before ablation. Although intracardiac-echocardiography may also be useful for detecting accessory LAAs on the MI, especially if they are larger than moderate size, it would likely be impossible to detect smaller ones. The cardiologists should always consider this possibility before performing MI ablation.

**Novel Teaching Points**

• Because of its fragile structure, accessory left atrial appendage (LAA) can cause cardiac tamponade during catheter ablation.
• When mitral isthmus (MI) ablation is planned, a careful review of preprocedure computed tomography scans is recommended in all cases to identify accessory LAA.
